# Current Trends in Neuromuscular Blockade, Management, and Monitoring amongst Singaporean Anaesthetists

**DOI:** 10.1155/2016/7284146

**Published:** 2016-10-13

**Authors:** Wendy H. Teoh, Thomas Ledowski, Phillip S. Tseng

**Affiliations:** ^1^Private Anaesthesia Practice, Wendy Teoh Pte. Ltd., Singapore; ^2^College of Anaesthesiologists, Singapore; ^3^School of Medicine and Pharmacology, University of Western Australia, Perth, WA, Australia; ^4^Anaesthetic Associates, Singapore

## Abstract

*Introduction*. This survey aimed to investigate the attitudes/practice pertaining the use, management, and monitoring of neuromuscular blockade amongst Singaporean anaesthetists.* Methods*. All specialist accredited anaesthetists registered with the Singapore Medical Council were invited to complete an anonymous online survey.* Results*. The response rate was 39.5%. Neuromuscular monitoring (NM) was used routinely by only 13.1% despite the widespread availability of monitors. 82% stated residual NMB (RNMB) was a significant risk factor for patient outcome, but only 24% believed NMB monitoring should be compulsory in all paralyzed patients. 63.6% of anaesthetists estimated the risk of RNMB in their own institutions to be <5%. 63.1% always gave reversal. Neostigmine was predominantly used (85.1%), with 28.2% using sugammadex at least sometimes, citing unavailability and high costs. However, 83.8% believed in sugammadex's benefits for patients' safety and >50% said such benefits may be able to offset the associated costs.* Conclusions*. There is a significant need for reeducation about RNMB, studies on local RNMB incidences, and strengthening of current monitoring practices and guidelines. Strategies are discussed. As NM monitors appear widely available and reversal of NMB standard practice, it is hopeful that Singaporean anaesthetists will change and strive for evidence-based best clinical practice to enhance patient safety.

## 1. Introduction

For decades, muscle relaxation has been used to facilitate tracheal intubation and operating conditions. Residual neuromuscular blockade (RNMB), defined as a train-of-four (TOF) (T4/T1) ratio of <0.9, in the postanaesthesia care (PACU) unit secondary to nondepolarizing muscle relaxants can result in potentially serious patient safety issues. RNMB has been reported to have a high incidence of 3.5 to 88% [1] and may cause demonstrable oropharyngeal dysfunction [2], adverse respiratory events [3], and prolonged stays in the PACU [4].

Despite a plethora of anaesthetic guidelines setting minimum monitoring standards for all anaesthetised paralyzed patients [5], there is widespread variability of practice and paucity of use of neuromuscular function monitors in Australia and New Zealand [6], USA and Europe [7], and Italy [8]. Surveys in Denmark [9], Germany [10], and United Kingdom [11] report that only 43%, 28%, and 10%, respectively, routinely use neuromuscular monitors of any kind. The introduction of sugammadex, a new reversal agent for aminosteroidal neuromuscular blocking agents (NMBA) has recently reignited the discussion around NMBA reversal and RNMB in Singapore. However, no reasonably recent knowledge exists about the incidence of RNMB and the practice of neuromuscular monitoring and NMBA reversal. We therefore embarked on this survey to investigate the current attitudes and practice relating to the management of neuromuscular blockade and monitoring patterns amongst Singaporean anaesthetists.

## 2. Methods

We sought ethical approval from the SingHealth Centralised Institutional Review Board (IRB) [application CIRB 2013/706/D] but the project was deemed exempt from IRB review. A list of all specialist anaesthetists registered with the Singapore Medical Council (SMC) was obtained and we invited them to participate in this anonymous online survey by emailing them a web link to complete the survey, through the Singapore Society of Anaesthesiologists and College of Anaesthesiologists database. The survey was available online for two months (21 August–21 October 2013), during which a reminder was sent out midway. The survey questions were designed by all investigators and the survey is constructed and distributed using “Survey Monkey,” a commercially available online survey vehicle, which was Internet Protocol address sensitive and only allowed one response per email address. Respondents were invited to complete the survey voluntarily and anonymously; no records of the email address were linked to any of the responses. The survey was also encrypted with a secure sockets layer to ensure privacy and message authentication.

The survey comprised 45 questions, designed to be answered over 10–15 mins, and examined the following in a multiple-choice-answer format: demographics of the anaesthetists (years of practice after specialist accreditation, nature of practice, whether government hospitals or private practice, and the size of their department), types of muscle relaxants available in their hospital, which relaxant they used for tracheal intubation and which for surgical relaxation, availability and use of neuromuscular transmission monitoring devices, attitudes and use of reversal agents, and perceptions and beliefs surrounding safe tracheal extubation criteria and residual neuromuscular blockade. There was also an opportunity to provide free text at the end of some questions.

Previously published email surveys of anaesthetists have reported a response rate of between 10 and 20% [6, 7]. We were hopeful for at least a 30% response rate of our specialist membership of 368 registered anaesthetists, that is, at least 110 responses. We defined residual neuromuscular blockade as a TOF <0.9 using any form of quantitative monitor. Quantitative monitors give an objective numerical response to the selected stimulus, by electromyography, acceleromyography, or kinemyography, whereas qualitative neuromuscular function monitors are those that require a subjective interpretation of the patients twitch response, such as a standard peripheral nerve stimulator.

## 3. Results

### 3.1. Response Rate

Of the 368 specialist anaesthetists registered with the Singapore Medical Council (SMC), eighteen had no emails listed or were found to have retired from active anaesthetic practice or were semiretired. Three were practicing abroad and hence excluded. Thus in August 2013, 347 email invitations were sent to anaesthetists practicing in Singapore with valid email addresses. Thirteen emails bounced due to recipients' full mailbox, recipient on annual leave with their automatic reply activated, or incorrect email addresses (that was subsequently rectified). Emails to these 13 practitioners were all successfully resent within the first week of the survey. At the end of the survey period, we received a total of 137 responses out of 347 (39.5% response rate).

### 3.2. Demographics

44.4% of respondents had more than 20 years of experience in anaesthesia, with 68.4% working in restructured (government funded) hospitals and 31.6% in private practice. The hospitals were generally well-staffed with 37.8% respondents coming from facilities employing >30 anaesthetists with postgraduate qualifications (registrar grade and above).

### 3.3. NMBA Availability and Use

Succinylcholine, atracurium, and rocuronium were reported as the most commonly available NMBA (96.2–98.5% availability), whereas mivacurium, pancuronium, vecuronium, and cisatracurium were unavailable to 50% or more of audited colleagues. In line with drug availability, 90.1% respondents used atracurium and 57.2% rocuronium to facilitate tracheal intubation. Succinylcholine was used at least sometimes by 70.2%. Anaesthetists polled believed that a deep neuromuscular block would facilitate surgery due to better operating conditions (64.6%) and prevention of sudden patient movement (42.3%) and potentially result in less pain (7.7%). In agreement with such beliefs, NMBA were not infrequently used during the maintenance phase of general anaesthesia (94.5% atracurium, 48.1% rocuronium, 3.8% pancuronium, and 1.53% mivacurium).

### 3.4. Monitoring

Though neuromuscular monitors were usually available to anaesthetic departments (95.8%), only 22.8% of respondents stated to have one monitor available for each theatre. More often (46.5%) one NM monitor was shared by 3 theatres. In contrast to the widespread availability of NM monitors, only 13.1% stated to monitor routinely. 60.3% of colleagues admitted that they would not change their current practice but still monitor only sporadically (<1/10 cases) even if more monitors were made available. The dominant reasons quoted for the decision not to monitor NMB were that monitoring was “unnecessary for experienced anaesthetists” (39.4%), “RNMB was irrelevant to the respondent's own practice” (15.2%), and “monitoring was too time consuming” (27.3%) and the belief that “monitoring was not sufficiently accurate to provide clinical benefit to patient safety” (36.4%). Correspondingly, only 29.7% believed that NM monitoring should be routine in all paralyzed patients, and an overwhelming 76.2% did not believe it should be part of minimum monitoring standards in anaesthetised paralyzed patients. This was in contrast to the fact that 62.5% of respondents stated the belief that clinical indicators were not sufficiently sensitive to identify patients with RNMB. However, about half (51.2%) of the respondents still reported to trust clinical signs of neuromuscular recovery such as a 5-second sustained head lift to be reliable predictors for the absence of significant RNMB.

### 3.5. Depth of Neuromuscular Block during Surgery

Of those who did monitor neuromuscular transmission intraoperatively, 63.4% stated they aim for the “traditional” depth of neuromuscular blockade (1-2 twitches in the TOF count) and only 15.9% reported to use deeper levels of muscle relaxation such as no twitch on either TOF or posttetanic count (PTC).

### 3.6. NMBA Reversal Timing

Most anaesthetists (62.8%) correctly identified that the TOF ratio should be >90% to extubate safely, but 35.3% thought TOF ratios of 70–90% were acceptable extubation criteria. 82% of all respondents stated that RNMB was a significant risk factor for patient outcome. The majority (72.7%) believed that RNMB negatively affected postoperative outcome and delayed discharge, thereby having a significant economic impact. The discordance between such beliefs and the lack of neuromuscular monitoring stated above may be due to the fact that the majority (63.6%) of anaesthetists estimated the risk of RNMB in their own institutions to be <5%. Both clinical signs and timing of the last muscle relaxant dose were quoted as the most relevant factors related to NMB reversal, with 63.1% always reversing at the end of surgery.

### 3.7. Reversal Drugs: Neostigmine

Neostigmine was predominantly used (85.1%), with only 28.2% respondents using sugammadex at least sometimes. Interestingly and in contrast to the overwhelming majority using neostigmine, 36.7% voiced the concern that neostigmine was associated with inadequate reversal of neuromuscular function. 47.7% were worried about its haemodynamic side effects, 45.9% quoted postoperative nausea and vomiting as an issue, and 33.1% were worried about respiratory events. Only 11.9% considered neostigmine a “clean” drug devoid of side effects. 50.9% of respondents would usually wait 3–5 min from the time neostigmine was administered to extubation, 30% waited 6–10 min, and only 5.1% would make the time interval from neostigmine administration to safe extubation dependent on the results of neuromuscular monitoring. 50.9% believed neostigmine produced reliable and rapid reversal at 4 TOF counts and 24.1% at 3 TOF counts. Most anaesthetists (60.5%) administered a dose of 2.5 mg neostigmine, whereas 24.6% were guided by a weight-based calculation of 0.05 mg/kg.

### 3.8. Reversal Drugs: Sugammadex

Though 83.8% believed in sugammadex's benefits for patients' safety and >50% stated that such benefits may be able to offset the associated costs, high costs and unavailability were still the mainly quoted reasons for not using sugammadex. The quoted main advantages of sugammadex were that it was more reliable (43.9%), was faster (31.4%), and had less side effects (30.5%) than neostigmine. 60% were not worried about any potential side effects of sugammadex whereas 29.6% were concerned about the risk of anaphylaxis, 11.1% patient coughing and sudden arousal, 7.4% interference with oral contraceptives, and 3.7% bleeding.

However, if sugammadex was available in their hospital without restrictions, 53.2% anaesthetists would use it more often in some patients and 16.5% in all patients. However, 16.5% would not use it at all. When asked if they or a close relative had to undergo surgery requiring muscle relaxation with rocuronium, 42.9% anaesthetists preferred to receive sugammadex as the reversal agent citing reliability, potential to reduce perioperative risk, and less side effects. 54.3% of respondents stated to prefer neostigmine because of its lower price.

## 4. Discussion

Our survey found that despite many anaesthetic guidelines calling for neuromuscular monitoring whenever NMBA are administered in the course of anaesthesia, the reality is that it is only routinely utilized by only 13.1% of respondents despite the widespread availability of monitors. Much of this may stem from old attitudes and the beliefs that monitoring was unnecessary for experienced anaesthetists, that it was insufficiently accurate to provide clinical benefit to patient safety (despite the overwhelming evidence in the literature [1]), and that monitoring would take up too much time. Most practitioners still base their decision to administer a reversal agent predominantly on clinical signs and timing of the last administered muscle relaxant dose, a practice not supported by any evidence.

This problem is however not a specific issue for Singaporean anaesthetists. A survey of 1440 Italian anaesthetists attending the 64th National Congress of the Italian Society of Anaesthesia, Intensive Care, Analgesia and Intensive Therapy (SIAARTI) found that 50% Italian anesthetists also used clinical signs (5-second head lift, tongue protrusion, and eye opening) to assess the recovery from neuromuscular blockade. The survey also found poor awareness of anaesthetists around the matter of RNMB and revealed their inability to identify even a significant degree of residual neuromuscular block [12].

In contrast to the common practice of using timing or clinical signs to exclude postoperative residual curarization, such tests have been shown to lack sufficient sensitivity [13]. In contrast, quantitative neuromuscular monitoring such as acceleromyography provides the only reliable tool to avoid residual curarization and to reduce the unnecessary use of reversal agents thus also reducing their side effects [13]. In this respect, we have identified a clear need for reeducation of anaesthetists in Singapore. All Singapore institutions should also aim to make one neuromuscular monitor available for each operating theatre and wherever possible replace older style qualitative monitors with newer quantitative ones.

We also found a discrepancy between a high knowledge-base and actual practice patterns amongst our colleagues. 82% respondents stated that RNMB was a significant risk factor for patient outcome and most believed RNMB negatively affected postoperative outcome and delayed discharge. Yet only 24% believed that NMB monitoring should be compulsory in all paralyzed patients. This is probably due to a majority (63.6%) of anaesthetists estimating the risk of RNMB in their own institutions to be <5%. Though this very likely constitutes a gross underestimation of the problem [1], no related data from Singapore currently exists to prove or reject the validity of such claims.

On a positive note, we found that a majority of Singaporean anaesthetists (63.1%) always reversed their patients at the end of surgery. In contrast, only 49% of anaesthetists in France consistently reversed their patients [14]. In the absence of appropriate monitoring, it is likely that administration of reversal agents as a standard may add a certain level of safety to an otherwise unsafe practice.

For NMBA reversal, neostigmine is undoubtedly most commonly used and cheaper, but due to its relatively nonselective pharmacodynamics, it is known to be associated with many side effects. Sugammadex, in contrast, is a selective antagonist indicated to reverse the effects of aminosteroidal NMBA by binding them in a 1 : 1 stable complex which is subsequently renally excreted. A multicentre, prospective, observational real-life study examining reversal outcomes of neostigmine versus sugammadex in 359 patients found that the time from reversal administration to TOF ratio 0.9 was significantly faster in the sugammadex group than in the neostigmine group (shallow block: 2.2 versus 6.9 min, resp., *p* < 0.0001; deep block: 2.7 versus 16.2 min, resp., *p* < 0.0001). Twenty minutes after reversal, TOF ratios of <0.9 only occurred in patients treated with neostigmine [15]. The latter is of utmost importance because sugammadex has been found to be not only faster than neostigmine, but also more reliable. It also enables anaesthetists to keep patients paralyzed throughout the anaesthetic, thus facilitating surgery. This opinion not just was expressed by anaesthetists in our survey but has also been reported in 2 prospective trials [16, 17].

Although sugammadex is considerably more expensive than neostigmine, its use may be advocated based on its safety and efficacy profile and as a means to prevent RNMB. As stated previously, RNMB is not just a common but also a dangerous condition which may lead to significant postoperative morbidity [1, 18, 19]. Indeed, increasingly more data has emerged corroborating the superiority of sugammadex over neostigmine as a reversal agent. A prospective audit confirmed fewer episodes of postoperative oxygen desaturation (15% versus 33%; *p* < 0.05) and showed reversal with sugammadex to be associated with the lowest rate of PONV [20]. A similar study in 1444 patients reported a likely reduction of postoperative pulmonary complications in elderly ASA physical status 3-4 patients when sugammadex versus neostigmine had been used [21].

Many anaesthetists claimed that the high cost for sugammadex was a reason for not using the drug at all or at least more often. Indeed, no prospective randomized controlled study has yet compared the real costs of sugammadex and neostigmine when “follow-on” costs (i.e., length of in-theatre or in-hospital stay) are factored into the calculation. However, a systematic review assessing the pharmacoeconomics of routine reversal with sugammadex compared with cholinesterase inhibitors (with cost assumptions based on average expenditures for staffing and drugs within the UK National Health Services) indicated that if reductions in recovery time associated with sugammadex in the trials are replicated in the operating theatre in routine practice, sugammadex would be cost-effective [22]. Simulation-based analysis into the efficacy of sugammadex has also showed an increase in additional cases over a few months without prolonging the working hours of staff, which may have an impact on procedural-related gross income [23]. When use of sugammadex was made available in daily clinical practice without restriction, the first-year experience at a major cancer centre found that although the total anaesthesia cost per case increased by €8.22, this was counterbalanced by faster patient turnover and reduced PACU times [24]. The authors concluded that the reduction of recovery times with sugammadex would reduce the incidence of prolonged extubation, resulting in faster turnover, and an increased patient's throughput. However, the achievable reduction of costs also depends on organizational factors, patient portfolio, and flexibility within the operating area [25].

A recent position paper on sugammadex use went so far as to advocate that “if a new drug is proven to be safer and more efficient than the one it is replacing, hospitals should consider the new drug and make it available, at least for selected patients or in situations at risk of severe complications. It is reasonable to hypothesize that, when discussing informed consent for elective procedures, patients and families may want to know if the admitting facilities have the superior agent available, and that the absence of such agent could create concerns and complains” [26]. This insightful advocacy introduces relevant medicolegal implications worthy of further analysis and may herald a 180° change in practice in the near future in Singapore.

Meanwhile, apart from utilizing neuromuscular monitoring whenever a NMBA is used to guide management and timely reversal, we propose an ongoing multimodal educational strategy. This involves ongoing internal lectures in each anaesthetic department or hospital, on the issue of neuromuscular monitoring and different monitoring strategies (i.e., quantitative versus qualitative), and on the issue of postoperative residual curarization (PORC) itself. We propose monitoring all patients who have received a NMBA intraoperatively for PORC in the PACU. If PORC is found, this could be fed back to the attending anaesthetist to review their practice. Additionally, most hospitals have quality assurance programmes or benchmarking process. The incidence of PORC could be included into these quality improvement processes for the PACU. Anonymous publication of the results in tandem with other benchmarking parameters (i.e., hypothermia and pain) as well as an audit before and after above-mentioned action would help to identify changes achieved and shortcomings.

## 5. Conclusion

This survey reveals a significant need for reeducation related to the matter of RNMB and reversal amongst Singaporean anaesthetists. However, it also shows a certain strength in the local system with neuromuscular monitors widely available and reversal of NMBA as standard practice. Therefore there is considerable hope that Singaporean anaesthetists will be able to change their standards related to the use of NMBA to reflect evidence-based best clinical practice. A similar change of practice has already been documented. From 1995 to 2004, monitoring of neuromuscular function in a French hospital increased from 2 to 60% and administration of reversal agents in the operating room increased from 6 to 42%. As a result, anaesthetists in this hospital found that their incidence of RNMB decreased from 62 to 3%, thereby confirming the impact of a simple change of practice on patient postoperative outcome [27].
